# Age Differences and Longitudinal Change in Exposure to Daily Stressors: Three Waves of Diary Data Across 20 Years

**DOI:** 10.1093/geroni/igab046.824

**Published:** 2021-12-17

**Authors:** Eric Cerino, Robert Stawski, Jonathan Rush

**Affiliations:** 1 Northern Arizona University, Flagstaff, Arizona, United States; 2 Oregon State University, Corvallis, Oregon, United States; 3 University of Victoria, Victoria, British Columbia, Canada

## Abstract

Exposure to daily stress is an important risk factor for healthy aging. We examined cross-sectional age-related differences and longitudinal aging-related change in stressor exposure across three waves of the National Study of Daily Experiences (N=2,914, M=51.53 years, SD=13.55, 56.35% Female) spanning 20 years. Exposure to six types of stressors (arguments, avoided arguments, work overloads, home overloads, network stressors, other) were obtained from telephone interviews over 8 consecutive days in waves conducted in ~1996, ~2008, and ~2017. Longitudinal analyses revealed declines in stressor exposure across 20 years (p <.01), driven by declines in arguments, work overloads, and network stressors specifically. Cross-sectional analyses indicated that older individuals reported stressors less frequently (p <.01), driven by decreases in arguments, avoided arguments, work overloads, and home overloads specifically. Rates of longitudinal decline did not depend on age at baseline. Results suggest that aging-related changes and baseline age differences inform daily stress trajectories in mid- and later-life.

